# Development and validation of a screener based on interRAI assessments to measure informal caregiver wellbeing in the community

**DOI:** 10.1186/s12877-018-0986-x

**Published:** 2018-12-13

**Authors:** Raquel S. D. Betini, John P. Hirdes, Nancy Curtin-Telegdi, Lisa Gammage, Jennifer Vansickle, Jeff Poss, George Heckman

**Affiliations:** 1 University of Waterloo, 200 University Avenue West, Waterloo, ON N2L 3G1 Canada; 2 Nucleus Independent Living, Oakville, ON L6H 6P5 Canada; 3 Hamilton Niagara Haldimand Brant Local Health Integration Network, Hamilton, ON L8J 0G5 Canada; 4 Schlegel Research Institute for Aging, 250 Laurelwood Dr, Waterloo, ON N2J 0E2 Canada

**Keywords:** Carer, Long-term care home, Screener, Home care, interRAI

## Abstract

**Background:**

Informal caregivers are invaluable partners of the health care system. However, their caring responsibilities often affect their psychological wellbeing and ability to continue in their role. It is of paramount importance to easily identify caregivers that would benefit from immediate assistance.

**Methods:**

In this nonexperimental cohort study, a cross-sectional analysis was conducted among 362 informal caregivers (mean age 64.1 years, SD ± 13.1) caring for persons with high care needs (mean age 78.6 years, SD ± 15.0). Caregivers were interviewed using an interRAI-based self-reported survey with 82 items covering characteristics of caregivers including key aspects of wellbeing. A factor analysis identified items in the caregiver survey dealing with subjective wellbeing that were compared against other wellbeing measures. A screener, called Caregiver Wellbeing Index (CWBI), consisting of four items with response scores ranging from 0 to 2 was created. The CWBI was validated in a follow-up study in which 1020 screeners were completed by informal caregivers of home care clients. Clinical assessments of the care recipients (*n* = 262) and information on long-term care home (LTCH) admission (*n* = 176) were linked to the screener dataset. The association between the CWBI scores and caregiver and care recipient characteristics were assessed using logistic regression models and chi-square tests. The reliability of CWBI was also measured.

**Results:**

The CWBI scores ranging from zero to eight were split in four ‘wellbeing’ levels (excellent, good, fair, poor). In the validation study, fair/poor psychological wellbeing was strongly associated with caregiver reports of inability to continue in their role; conflict with family; or feelings of distress, anger, or depression (*P* < 0.0001). Caregivers caring for a care recipient that presented changes in behavior, cognition, and mood were more likely to present fair/poor wellbeing (*P* < 0.0001). Additionally, caregivers with high CWBI scores (poor wellbeing) were also more likely to provide care for someone who was admitted to a LTCH (OR 3.52, CI 1.32–9.34) after controlling for care recipient and caregiver characteristics. The Cronbach alpha value 0.89 indicated high reliability.

**Conclusion:**

The CWBI is a valid screener that can easily identify caregivers that might benefit from further assessment and interventions.

## Introduction

Informal caregivers, defined here as family members or friends caring for an adult with a chronic or disabling condition, are an integral part of the home and community care system in many countries. The care provided by informal caregivers is paramount for supporting those in need to remain at home for as long as possible and to avoid institutional care [1]. Additionally, the provision of unpaid care results in substantial cost savings for the health system by off-setting paid home care service and reducing long term care home admissions [2].

However, informal caregivers are often at risk of poor health outcomes such as depression and physical pain, especially when caring for persons with high care needs [3–5]. The ability of informal caregivers to continue in their role is often threatened when they face increasing demands to provide care for individuals with complex health issues. Indeed, the sustainability of the home care system relies on the capacity of informal caregivers to function effectively in their role as providers of support.

Psychological wellbeing, defined as ‘the state of equilibrium or balance that can be affected by life events or challenges’ [6] is often negatively affected by physical and psychological challenges of caregiving [5, 7]. This is a worrisome issue because poor psychological wellbeing has been associated with poor health and shorter survival in older adults [8]. On the other hand, caregiving may also bring positive experiences that may have a protective role in health maintenance [9–11].

A major challenge for the health system is to measure caregiver wellbeing to identify those at risk of poor quality of life (i.e., psychological, physical, social, material wellbeing) [12] or health declines in order to provide appropriate supports to address their needs. Most available instruments to screen caregivers are impractically long for initial assessment, lack cut-offs points for intervention, or lack validity and reliability [13–16]. Moreover, most caregiver assessments are not designed to be compatible with assessment tools already in widespread use for assessment of home care clients.

This study uses established interRAI assessment systems [17]; specifically, the RAI Home Care (RAI-HC), the interRAI Community Health Assessment (interRAI CHA), the interRAI/Kendal Corporation Collage “Wellness” Assessment, and the interRAI Quality of Life Self-report Surveys for Home Care/Community Living, Senior Housing, and Mental Health [18]. These assessments were developed by interRAI, an international group of researchers and clinicians with a focus on improving care for vulnerable populations (e.g., child and adult mental health, older adults in the acute care setting, residents in long-term care, adults in acute and longer-term community care). The RAI Home Care Assessment is a validated multidimensional assessment for adults [17] and is currently used in a number of jurisdictions in North America (Canada and multiple states in the U.S.), Europe (Italy, Switzerland, Finland, Estonia, etc.), and Asia/Pacific Rim (Hong Kong, Singapore, Japan, Australia, New Zealand) [19]. This assessment includes items related to various domains: health, function, social support, diagnosis, services use etc. describing around 300 characteristics [20, 21]. Risk scales are derived from calculations using items in the assessment (e.g., Cognitive Performance Scale [22, 23], Depression Rating Scale [24], Method for Assigning Priority Levels (MAPLe) [25, 26]).The interRAI CHA is based on the interRAI HC assessment but is intended to be used by community support organizations to assess potential clients that are looking for day activities, meals, and personal support [26]. The interRAI CHA is a modular assessment system and includes a core assessment (for all clients) and four supplements that are used to evaluate specific problems in depth as needed (functional, mental health, assisted living and deafblind assessments) [18]. While not as extensively used as the RAI-HC or its newer version, the interRAI HC assessment, the interRAI CHA has been used in research or mandated for use in various jurisdictions (e.g., Finland, Hong Kong, New Zealand, Australia, Israel, and Ontario, Canada). The interRAI CHA core items are similar to those in the RAI-HC and risk scales can also be derived from the interRAI CHA [26].

Both assessments highlight issues related to functioning and quality of life for community-residing individuals and have common items that were used to describe and investigate the characteristics of the care recipient. Health care providers (e.g., social workers, nurses) are trained to perform these assessments using information obtained from the client, their caregiver, and medical records. Using these well-established assessments in the development of a caregiver screener allows for more accurate and representative evaluation of associations between caregiver and care recipient characteristics and needs.

The interRAI/Kendal Corporation Collage “Wellness” Assessment is a self-report assessment that addresses issues related to a person’s psychosocial, cognitive, health status, and daily functioning based on items from interRAI community assessments. The interRAI Quality of Life Self-report Surveys for Home Care/Community Living, Senior Housing, and Mental Health and are self-report surveys that address a number of issues, including, for example, self-determination, mood, social participation and community involvement, social ties, and belonging. These self-report assessments provided the researchers with established interRAI-based items on which to develop a self-report survey for caregivers.

Studies reviewing caregiver assessments point to lack of 1) evidence on psychometric properties, 2) information on sensitivity to change for several caregiver measures [1–3], and 3) theoretical approach to explain the concept behind the development of the assessment [27–29]. Also, because most assessments include multiple domains [15], they are not suitable to be used for screening purposes and the sensitivity to change of the instrument may be low if the instrument mixes items that represents different domains (e.g., objective and subjective burden) that could be affected differently by interventions. In other words, specific measures should be used to identify changes within significant caregiver outcomes (e.g., depression, psychological wellbeing) [30]. While the vast majority of screeners reported in caregiver research measure caregiver burden and other negative aspects of caregiving [31–33], this study develops and validates a measure of caregiver psychological wellbeing that is influenced by both negative and positive experiences. Caregiver psychological wellbeing is a valuable measure to be assessed before and after interventions as it reflects how caregivers are coping with caregiving challenges or stressful situations [34, 35].

A short screener that captures key aspects of a caregiver’s psychological wellbeing would help organizations identify caregivers that would benefit from immediate assistance and further detailed assessment. Such a screening and further assessment, if warranted, could identify areas of need (e.g., depression), and allow for timely implementation of interventions to support the caregiver and evaluate changes in the caregiver’s wellbeing following implementation of interventions for the caregiver. The objective of this study was to develop and validate a screener, compatible with established care recipient assessments (i.e., interRAI suite) [17, 36], to measure caregiver psychological wellbeing and identify caregivers at risk of adverse outcomes.

## Methods

This study involved two stages. The first stage, the development of the screener, is represented by a nonexperimental longitudinal cohort study that includes a baseline family caregiver assessment at program admission and one follow-up assessment 6 months later. The second stage, the validation study, is also a nonexperimental longitudinal cohort study where assessment data was collected simultaneously for the caregiver and care recipient. Information on care recipient discharge from home care to long term care home was also obtained for this study.

### Screener development

As an initial step, a Caregiver Survey was developed using items from the interRAI/Kendal Corporation Collage “Wellness” Assessment and the interRAI Quality of Life Self-report Surveys for Home Care/Community Living, Senior Housing, and Mental Health. All items from the Wellness Assessment and the Quality of Life self-report surveys were considered for the interRAI Caregiver Survey, with or without modifications. Over the course of a number of revisions, a pilot interRAI Caregiver Survey was finalized and approved by interRAI with a total of 82 items covering a range of domains including demographic information, physical and emotional health, caregiving responsibilities, well-being, supports, and quality of life. The interRAI Caregiver Survey was intended to capture characteristics of a caregiver, with the goal of identifying those characteristics that were most pertinent to caregiver wellbeing.

#### Derivation sample

The informal caregivers in this study were participants of the Caregiver Recharge Service (CRS), a respite program for informal caregivers offered through the Mississauga Halton Local Health Integration Network (MH LHIN), in Ontario, Canada. The CRS was established to provide respite to caregivers by offering in-home assistance with activities they usually perform with the goal of reducing caregiver strain, improving caregiver wellbeing and increasing their ability to continue caring activities.

In order to participate in the CRS program, two sources of information were used. The Caregiver Strain Index (CSI), an assessment to identify caregivers experiencing strain [37, 38], was completed by the caregiver. The other source of information was derived from the care recipient’s interRAI CHA or RAI-HC assessment [17, 23], specifically the Method of Assigning Priority Levels (MAPLe) score. MAPLe is a decision-support tool with scores ranging from 1 to 5 used to identify community care recipients who have the most urgent need for care [25]. It includes items dealing with behaviour issues, cognitive and activity of daily living impairment, falls, and other types of health problems that have been associated with caregiver distress and long-term care home (LTCH) admission [25]. Caregivers eligible for the program included those who scored 9 or higher on the CSI assessment (maximum score of 13), indicating high strain, and were caring for a care recipient with high needs as indicated by a MAPLe score of 4 or 5 or receiving palliative care.

#### Recruitment

Participants in the study were recruited from three health service providers (HSP) involved in the Caregiver Recharge program: Links2Care, Home Instead, and Able Living. Staff from these organizations introduced the study to 560 caregivers during their first visit by providing the caregiver with a letter from the researchers explaining the study and then obtaining written consent to participate in the study from those caregivers who agreed to do so. Once consent was obtained, the research team was provided with the signed consent form, the caregiver’s phone number, and best time to contact them. These organization also provided the research team with care recipient assessments (interRAI CHA or RAI-HC) and discharge summary reports.

Trained volunteers of the research team interviewed caregivers on the phone using the the interRAI Caregiver Survey within 1 week of receiving the consent. A total of 362 caregivers (65% of 560 that were approached) completed the caregiver survey on the phone at baseline (i.e., CRS admission) between September 2013 and September 2015. Initially, caregivers cited lack of available time to complete the survey. In order to increase response rate and minimize bias related to inclusion of ‘less busy’ caregivers, the study team members offered more flexible interview times (e.g., before 8 AM, after 6 PM).

A total of 169 caregivers completed the survey at least 6 months after the first assessment. Most caregivers that did not complete the follow-up survey were no longer participating in the CRS program because the respective care recipient died or was admitted to LTCH. Also, some caregivers were unavailable to complete the survey (e.g., did not answer the phone or reported to be busy). The data on the Caregiver Survey was entered by the interviewer in an excel spreadsheet after the phone call. The care recipient assessments (i.e., interRAI CHA or RAI-HC) were sent electronically to the research team using standard and approved methods for secure transmission of data.

The RAI-HC is used by the home care provider of the MH LHIN to assess home care clients for determining needs and home care services. The majority of the referrals to the Caregiver Recharge program come from this organization (i.e., 60%) and as such, the care recipient’s RAI-HC was used in the study. However, as part of routine practice, care recipients in the Caregiver Recharge program that did not have a RAI-HC assessment were assessed using the core and functional supplement of the interRAI CHA by a trained member of the agency staff. Thus, these assessments were also available to the research team.

Care recipient assessments performed by an organization more than 1 month before there was a referral for the caregiver respite program were excluded from the study. Thus, only 262 caregiver surveys that were completed close to the care recipient assessment were included in the analysis to ensure that both assessments reflected the caregiving background situation. All palliative clients were also excluded from the analysis due to the low number of available linked caregiver assessments (*n* = 20). Full ethics clearance for this study was granted by the Office of Research Ethics, University of Waterloo (#18982).

#### Statistical analysis

The aim of this study was to develop a brief caregiver screener to measure psychological wellbeing of caregivers based on indicators of psychological wellbeing derived from the pilot interRAI Caregiver Survey. For identifying the items that best represent psychological wellbeing, an exploratory factor analysis (EFA) was used to examine the association between the items representing different domains in the survey (e.g., physical and mental health, social supports) and an underlying construct representing caregiver subjective wellbeing.

For data preparation before the factor analysis, results of frequency analyses of 531 caregiver surveys (first assessment: 362; reassessment: 169) were examined and a total of 24 items were removed based on low variability (that is, at least 85% of the sample sharing the same response for the same question). For example, some of the variables removed due to lack of variability were: ‘caregivers did not receive help with bathing’, ‘caregiver diagnosis of dementia’, ‘caregiver diagnosis of Alzheimer’s disease’, ‘caregiver reported hip fracture’, ‘caregiver reported any fracture’, ‘caregivers taking care of an adult family member or friend’. In addition, 14 items were removed because they were related to demographic information (e.g., gender, age, living arrangement, primary language, full time/part time job) leaving 44 items for analysis. According to Costeloo and Osborne (2005) a subject to item ratio greater than 10:1 (i.e., 440 sample size) is considered appropriate for exploratory factor analysis [39].

The random distribution of missing values was evaluated through the missing data pattern yielded by the procedure “MI” in SAS. The use of an imputation method to replace missing values with the mean of the variable was performed after determining the random distribution of missing values. Lastly, the scoring of reverse-phrased items was inverted.

A Kaiser-Meyer-Okin (KMO) score of 0.86 indicated that a factor, or factors, could be extracted from the dataset [40]. The Barlett’s Test of Sphericity was performed to ensure that the dataset was appropriate for extracting factors. The null hypothesis (no factors) was rejected (*P* < 0.0001) confirming that factors could be extracted.

A parallel analysis (PA), a method based on Monte Carlo simulation, was performed to obtain the number of factors to be extracted. This method overcomes some of the limitations of the Kaiser’s method, or mineigen greater than 1 criteria, such as misleading results due to sampling error [41]. A SAS Macro for parallel analysis was used [42] including 100 iterations. Oblique rotation was chosen as the most appropriate type of rotation for this analysis because it allows for correlation among factors [43], aligning with evidence on the interactions between variables representing different aspects of caregiver’s life [44–46].

The factor analysis was performed after removing items with factor loadings lower than 0.35. After identifying the factor representing the psychological wellbeing construct, the second step of the analysis was to confirm whether items with loading higher than 0.35 that were identified as part of factor 3 (psychological wellbeing) were correlated with measures that one would expect to be associated with psychological wellbeing – i.e., measure of strain (scores of Caregiver Strain Index) and depression. The latter is an item in the caregiver survey which states: ‘caregiver is currently receiving treatment or being monitored for depression’. For this analysis, scores from 1 to 5 were assigned for each response of the seven items in ‘Factor 3’ with high loading (bolded in Table 2), with higher values corresponding to higher frequency of the expressed feeling (e.g., never/not in the last 3 days, but do have the problem/1 day/ 2 days; and every day in the past 3 days). Only the interRAI-based Caregiver Surveys completed at baseline with a CSI completed within a 90 days interval between these assessments were used for this analysis (average time between CSI and survey = 37 days; SD 20.2, *n* = 286) to ensure that the CSI assessment score reflected the status of the caregiver at the time of the survey.

In the third step, a series of chi-square tests was performed to evaluate the association of the sum of different combinations of the selected items scores from the previous step with 1) CSI scores converted to a binary variable and 2) the ‘depression item’.

The next section describes the evaluation of screener cut-offs and validation of the Caregiver Wellbeing Index (CWBI) screener using two datasets respectively: 1) derivation dataset and 2) validation study dataset.

#### Evaluation of screener cut-offs

Logistic regression models were used to evaluate if the CWBI screener cut-off points were associated with different likelihood of caregiver related outcomes, including: 1) physician visits; 2) self-reported health; 3) personal outlook; and 4) the caregiver reporting that the care recipient would be better off elsewhere. Personal outlook is defined here as “unique patterns of thinking, feeling and interacting with ourselves” [47], related but not similar to psychological wellbeing. These outcomes were chosen because they are related to different domains of caregiver’s lives that are often related to subjective wellbeing [8, 48].

The dependent variable responses from the interRAI Caregiver Survey were collapsed as follows: 1) appointment with doctor or nurse practitioner in the last 90 days (any vs. none); 2) self-reported health (fair/poor vs. excellent/good); 3) caregiver reports that ‘on the whole my life is good’ (never, rarely or sometimes vs. most of time or always); and 4) caregiver reports that they ‘believe that their care recipient would be better off elsewhere’ (yes vs. no). All surveys completed at intake were included in this analysis (*n* = 362). The association of the screener score levels with the caregiver outcomes was evaluated after controlling for age and gender, which were included as covariates in the models. Both covariates were chosen because of evidence showing that caregiver experiences and related outcomes may differ according to age [49–51] and gender [51–57].

In the last step, chi-square analyses examined whether CWBI levels were also associated with other variables related to caregiver subjective wellbeing such as ability to continue caring and loneliness [58]. The reliability of the screener was examined using standardized Cronbach alpha value.

### Screener validation (based on follow-up study)

A test of validity of the CWBI was performed using informal caregiver and respective care recipient information derived from home care clients in a subsequent pilot study. Predictive validity of the screener was examined by evaluating whether the CWBI scores predicted care recipient LTCH admissions.

A total of 1020 primary informal caregivers of long-stay home care clients in Hamilton Niagara Haldimand Brant Local Health Integration Network (HNHB LHIN) completed the CWBI screener as part of routine assessments between April 01st, 2016 and March 31st, 2017. Only the CWBI completed within 3 months of the RAI-HC assessment for the care recipients with MAPLe 3 to 5 living in a private home or apartment were included in the dataset.

The RAI-HC assessments are available to the researchers based on license agreements with interRAI. To guarantee the anonymity of the data, the dataset did not include any personal identifiers. Information on care recipient institutionalization was obtained from the Client Health and Related Information System (CHRIS), a web-based care recipient management system that collects information on home care client admission and discharge.

Validation of the screener was performed by evaluating the association (i.e., chi-square test) between the CWBI scores and the caregiver related information that is captured in the RAI-HC items completed during the care recipient assessment that provides information on areas of help and caregiver status. These items were selected for the analysis because of their relevance to the caregiving experience. The items on the ‘caregiver status’ section report information on 1) caregiver ability to continue in their role; 2) conflict with family; and 3) feelings of distress, anger, or depression. The information for these items is obtained from multiple sources (e.g., the care recipient, the caregiver, other care providers, the assessor’s observations). The recorded responses are based on the assessor’s clinical judgement that best summarizes all of the information about the presence or absence of caregiver distress. As such, the responses do not necessarily represent only the caregiver’s perception. This is a key difference to the caregiver screener which is a ‘self-reported’ item that specifically reflects the caregiver’s perception of their wellbeing.

Chi-square test, a method used to examine association between variables and test validity [59], was used to examine the association between CWBI scores and care recipient characteristics that affect the demand of care provided by caregivers often altering their caregiver psychological wellbeing [60–62]. For example, items contained in the RAI-HC and interRAI CHA assessments that describe cognitive impairment, bowel and urinary incontinence, and behavioural symptoms.

Logistic regression models were used to examine whether CWBI scores predict LTCH admission of the care recipient, after controlling for covariates such as caregiver and care recipient age and gender, caregiving dyad co-residence, and care recipient health care needs.

The reliability of the screener was also measured in this validation study using standardized Cronbach alpha value.

The following section provides the sample description and results of the analysis performed in the study for the development of the screener.

#### Caregiver and care recipient samples

Table 1 provides the main characteristics of the caregivers by age. The majority were female, 52% were over 64 years old, and most in this group were spouses of the care recipient. Younger caregivers were more likely to be involved in full or part time work and caring for children in addition to an older adult in the family compared to the older caregivers. A higher proportion of younger caregivers (< 65 years old) experienced financial challenges than older caregivers (65+).

**Table 1:** Caregiver demographics, financial status, and health conditions on admission to the Caregiver Recharge Services

Age group (years)	< 45	45 to 64	65 to 74	75+
	*% (n)*	*% (n)*	*% (n)*	*% (n)*
	10.6 (28)	37.7 (99)	24.8 (65)	26.7 (70)
Caregiver Strain Index				
9, 10, 11	67.8 (19)	62.6 (62)	76.8 (50)	88.6 (62)
12, 13	32.2 (9)	37.4 (37)	23.2 (15)	11.4 (8)
Gender				
Female	69.6 (11)	78.0 (68)	64.7 (49)	64.2 (43)
Primary Language				
English	64.3 (18)	61.6 (61)	67.6 (44)	67.1 (47)
Caregiver Relationship				
Spouse	7.14 (1)	10.3 (8)	73.6 (39)	90.0 (45)
Child	71.2 (10)	75.6 (59)	12.2 (10)	3.66 (3)
Other	21.4 (3)	14.1 (11)	7.55 (4)	4.0 (2)
Paid Employment				
Part time or full time	27.3 (3)	33.9 (18)	6.78 (4)	2.99 (2)
Caring for				
Adult only	42.8 (12)	55.5 (55)	16.9 (54)	7.14 (65)
Adult and child	39.3 (11)	38.4 (38)	13.8 (9)	2.86 (2)
Economic Trade-offs^a^				
Yes	23.1 (6)	30.9 (30)	24.6 (16)	7.46 (5)
Physical Health^b^				
Diabetes	17.8 (5)	11.1 (11)	24.6 (16)	15.9 (11)
Depression	10.7 (3)	19.2 (19)	16.9 (11)	8.70 (6)
Cancer	3.57 (1)	6.01 (6)	7.69 (5)	11.6 (8)

In some cases, the number of cases is less than the full sample size because missing data are excluded

^a^In the last 30 days, have you made trade-offs among purchasing: adequate food or shelter, clothing or prescribed medications, sufficient home heat or cooling, necessary health care or home care due to limited funds?

^b^Currently receiving treatment or being monitored for the health condition

More than half of care recipients were married (55%) and 35% were widowed. The mean age of care recipients in this study was 78 (*n* = 260, SD 15.1), 51% were female, 58% had a dementia diagnosis, 41% had a MAPLe score of 5, and 55% of the care recipients needed at least extensive assistance for completion of activities of daily living.

### Screener development - results

#### Factor analysis

As shown in Table 2, a factor representing psychological wellbeing (factor 3) was identified in addition to two factors representing other aspects of caregiver life: physical health (factor 1) and psychosocial support (factor 2). The correlation between factors ranged between 0.31 and 0.50, with highest values between psychosocial support and psychological wellbeing factors (0.50). The standardized Cronbach values were 0.77, 0.83 and 0.76 for factors 1, 2 and 3 respectively, indicating acceptable or good internal consistencies.

**Table 2:** Factor structure matrix rotated with oblique rotation

Items	Item	Factor 1	Factor 2	Factor 3
Dizziness	*b9a*	***0.44***	− 0.96	0.16
Unsteady gait	*b9b*	***0.55***	0.03	−0.13
Pain	*b9d*	***0.75***	−0.03	−0.08
Difficulty falling or staying asleep	*b9h*	0.32	−0.14	***0.39***
Self-rated health	*b1*	***0.44***	0.22	−0.03
Tiredness	*b8*	***0.58***	0.04	0.04
Highest level of pain intensity	*b10*	***0.75***	−0.04	−0.05
Shortness of breath	*b12*	***0.42***	0.05	−0.04
Little or no pleasure in things that normally enjoy	*b16*	0.14	0.09	***0.40***
Anxious, restless, or uneasy	*b17*	0.28	−0.08	***0.51***
Sad, depressed, or hopeless	*b18*	0.18	0.07	***0.57***
Overwhelmed by your relative/friend’s illness	*c2b*	0.14	0.03	***0.52***
Good relationships with family and friends	*e1a*	−0.05	***0.61***	−0.10
Have people can count on	*e1b*	−0.05	***0.58***	0.00
Hopeful about future	*e1c*	−0.01	***0.45***	0.30
Feel good about self	*e1d*	0.14	***0.55***	0.13
Life is good	*e1e*	0.01	***0.59***	0.22
Valued and respected by others	*e1f*	−0.01	***0.64***	0.01
Play important role in people’s lives	*e1g*	0.06	***0.60***	−0.20
Feel part of community	*e1h*	0.05	***0.38***	0.19
Participate in meaningful activities	*e1i*	− 0.03	***0.37***	0.25
Get the health services needed	*e1j*	0.02	***0.51***	−0.08
Can get help right away	*e1k*	−0.04	***0.43***	0.04
Can be alone when wish	*e1o*	−0.22	−0.02	***0.57***
Can go out on the “spur of the moment”	*e1p*	−0.16	− 0.03	***0.59***
Variance explained by each factor (%)		3.74	4.29	3.92

The results of the correlations between each of the items with high loading (bolded items of ‘Psychological wellbeing’ factor) and 1) strain measure (i.e., CSI) and 2) ‘depression item’ showed that only the following items were statistically correlated with both the CSI scores and the ‘depression items’*(P* < 0.05) that ask: ‘In the last three days, how often have you felt: 1) little or no pleasure in the things you normally enjoy, 2) anxious, restless, or uneasy, 3) sad, depressed or hopeless, and 4) overwhelmed by your relative/friend’s illness?’. All selected questions represent ‘caregiver feelings’ experienced in the 3 days prior the survey. The following five possible responses were: 1) never; 2) not in the last 3 days, but do have the problem; 3) on 1 day; 4) on 2 days; and 5) every day in the past 3 days.

The best distribution of the sum of the four item scores associated with CSI scores (9, 10, 11 vs. 12, 13) and the ‘depression item’ was obtained when assigning a score of zero for the responses 1) never and 2) not in the last 3 days, but do have the problem; a score of ‘1’ for responses 3) 1 day and 4) 2 days; and a score of ‘2’ for the response 5) every day in the past 3 days.

The resulting summary score of the four questions of the CWBI screener ranged from 0 to 8 with cut points zero, 1 to 3, 4 to 6, and 7 to 8 obtained after evaluating the frequency distribution of the CWBI screener scores and CSI.

#### Logistic regression

The association between the CWBI and caregiver related variables was assessed using logistic regression models that included age and gender of the care recipient as covariates in the model.

Table 3 shows that caregivers with higher CWBI scores were more likely to have had doctor/nurse appointments in the last 90 days and poor or fair self-reported health. They were also more likely to report that their lives were never, rarely, or only sometimes good and that they believed the care recipient ‘would be better off elsewhere’. The CWBI predicted all outcomes after controlling for caregiver age and gender.

**Table 3:** Outcomes associated with Caregiver Wellbeing Index (CWBI)^a^

Independent variable	Parameter estimate (SE)	Odds ratio (95% CI)	*P* value	C statistict
Caregiver - Doctor/nurse appointment (At least one appointment in the last 90 days)^a^				
CWBI (1–3 vs. 0)	− 0.06 (0.33)	1.93 (0.48–1.78)	0.83	0.68
CWBI (4–6 vs. 0)	1.13 (0.39)	3.10 (1.44–6.67)	0.003	
CWBI (7–8 vs. 0)	0.98 (0.49)	2.67 (1.00–7.10)	0.048	
Age group (≥60 and < 75 vs. < 60)	0.17 (0.30)	1.19 (0.66–2.14)	0.56	
Age group (≥75 vs. < 60)	1.34 (0.39)	3.84 (1.77–8.34)	0.0006	
Gender (female vs. male)	0.13 (0.31)	1.15 (0.62–2.10)	0.65	
Caregiver Self-reported health (Poor or Fair)^a^				
CWBI (1–3 vs. 0)	0.41 (0.34)	1.51 (0.77–2.97)	0.22	0.67
CWBI (4–6 vs. 0)	1.34 (0.35)	3.82 (1.91–7.63)	0.0001	
CWBI (7–8 vs. 0)	1.30 (0.44)	3.68 (1.54–8.83)	0.0035	
Age group (≥60 and < 75 vs. < 60)	0.39 (0.29)	1.48 (0.83–2.64)	0.17	
Age group (≥75 vs. < 60)	0.74 (0.32)	2.09 (1.10–3.96)	0.02	
Gender (female vs. male)	0.06 (0.28)	1.07 (0.61–1.89)	0.81	
Caregiver reports ‘On the whole, my life is good’ (Never, rarely and sometimes)^a^				
CWBI (1–3 vs. 0)	0.43 (0.44)	1.54 (0.64–3.69)	0.33	0.73
CWBI (4–6 vs. 0)	1.58 (0.42)	4.87 (2.12–11.2)	0.0002	
CWBI (7–8 vs. 0)	2.60 (0.53)	13.53 (4.80–38.1)	< 0.0001	
Age group (≥60 and < 75 vs. < 60)	−0.04 (0.32)	0.95 (0.50–1.81)	0.88	
Age group (≥75 vs. < 60)	−0.30 (0.39)	0.74 (0.34–1.59)	0.43	
Gender (female vs. male)	0.02 (0.33)	1.02 (0.53–1.97)	0.95	
Caregiver believes that care recipient would be better off elsewhere (Yes)^a^				
CWBI (1–3 vs. 0)	0.65 (0.44)	1.92 (0.81–4.55)	0.13	0.64
CWBI (4–6 vs. 0)	0.84 (0.44)	2.33 (0.97–5.56)	0.05	
CWBI (7–8 vs. 0)	1.44 (0.51)	4.24 (1.54–11.6)	0.005	
Age group (≥60 and < 75 vs. < 60)	0.75 (0.35)	2.14 (1.07–4.25)	0.03	
Age group (≥75 vs. < 60)	0.43 (0.40)	1.54 (0.70–3.40)	0.27	
Gender (female vs. male)	0.23 (0.33)	0.79 (0.41–1.53)	0.48	

^a^Interaction between age and CWBI was not significant (*P* > 0.05). Age group and gender are reported for care recipients. Caregiver age and gender were not significant in the models and not reported in the table

The standardized Cronbach value calculated for the CWBI was 0.75, indicating that this algorithm has an acceptable internal consistency [63].

**Fig. 1:**
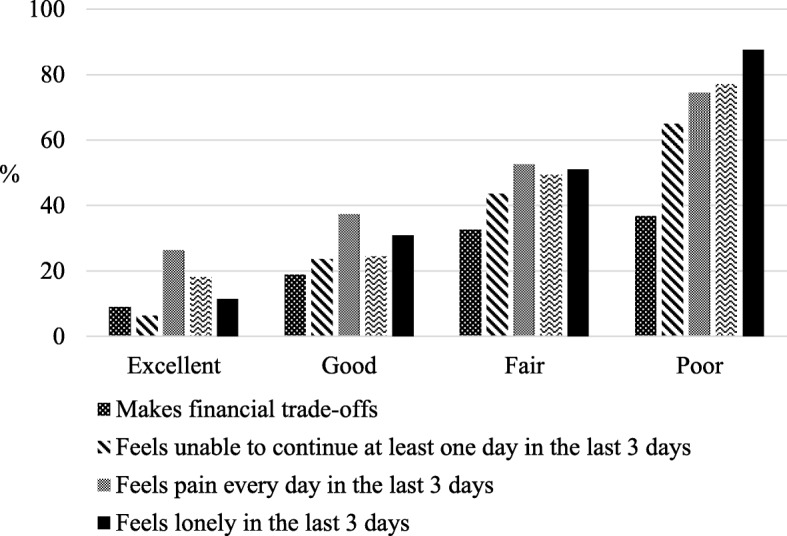
Caregiver reports: 1) financial issues; 2) inability to continue caring; 3) physical pain every day; and 4) loneliness by CWBI levels

**Fig. 2:**
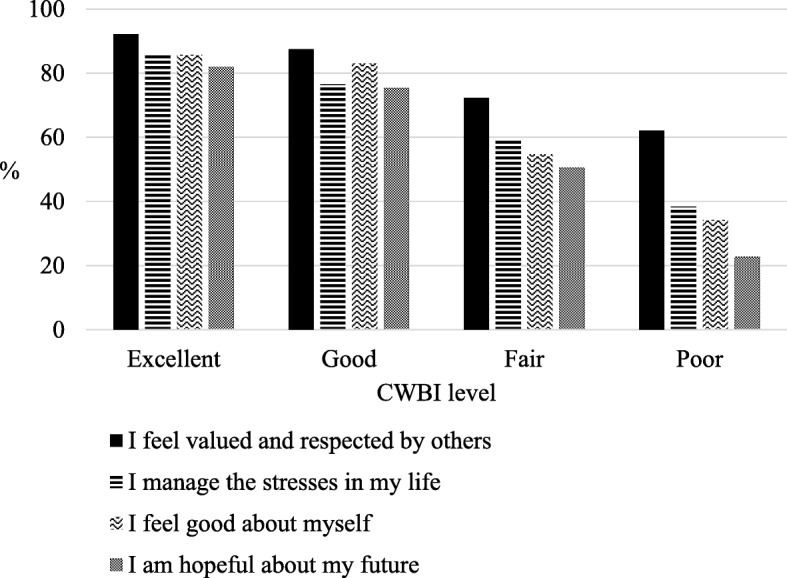
Caregivers report that ‘always, or most of the time’: 1) feel valued and respected by others; 2) manage the stresses in their life; 3) feel good about themselves; 4) are hopeful about their future

As shown in Fig. 1, other caregiving issues were also related to caregiver poor wellbeing (higher CWBI scores). In contrast, Fig. 2 shows that positive characteristics were related to excellent/good self-reported caregiver wellbeing (all Chi square values had *P* < 0.0001).

## Validation study - results

The age groups ‘younger than 45’, ‘45 to 64’, ‘65 to 74’ and ‘75 or older’ represented 5, 34, 21 and 39% of the informal caregivers in the follow-up study (*n* = 1020). More than half (54%) were spouses or partners and 36% were children of the care recipient. A total of 85% of all caregivers resided with their respective care recipient. More than half of care recipients were married (62%) and 32% were widowed. The mean age of care recipients in this study was 79 (n = 1020, SD 12.7), 54% were females, 37% had a dementia diagnosis, and 49% of the care recipients needed at least extensive assistance for completion of activities of daily living. Proportions of care recipient with MAPLe 3, 4 and 5 were 36, 38 and 26%, respectively.

There was a strong association between the CWBI scores with the following binary caregiver items in the RAI-HC: 1) caregiver is unable is continue caring activities, 2) primary caregiver is not satisfied with support provided by family and friends and 3) primary caregiver expresses feelings of distress, anger, or depression (Table 4). In addition, higher CWBI scores were associated with caregiver lack of willingness (with ability) to increase emotional support to the care recipient.

**Table 4:** Caregiver and care recipient characteristics in the RAI-HC by Caregiver Wellbeing Index (CWBI) levels

		Caregiver Wellbeing Index levels				
		Excellent	Good	Fair	Poor	
*Primary caregiver reports*	Total n	% (n)	% (n)	% (n)	% (n)	
Feeling unable to continue	387	14.3 (22)	28 (42)	38.2 (125)	50.9 (198)	<.0001
Conflict with family	189	3.25 (5)	17.3 (26)	18.3 (60)	25.2 (98)	<.0001
Feeling distress, anger, depression	662	20.1 (31)	53.3 (80)	73.1 (239)	80.2 (312)	<.0001
Feeling unable to increase emotional support	647	51.3 (79)	54.7 (82)	66.1 (216)	69.4 (270)	.0006
*Care recipient symptoms*						
Worsening of decision making^a^	444	29.8 (46)	28.7 (43)	42.8 (140)	55.2 (215)	<.0001
Worsening in communication^a^	254	12.9 (20)	14.7 (22)	26.6 (87)	32.1 (125)	<.0001
Changes in behavioural symptoms^a^	170	3.90 (6)	9.33 (14)	17.4 (57)	23.9 (93)	<.0001
Mood decline^a^	310	10.4 (16)	23.3 (35)	29.6 (97)	41.6 (162)	<.0001
MAPLe 5	265	12.3 (19)	15.3 (23)	29.6 (97)	32.4 (126)	

^a^compared to status of 90 days ago

Higher CWBI scores were strongly associated with the presence of the following behavioural symptoms of the care recipient: wandering (*P* < 0.05), verbal abusive (*P* < 0.0001), and resists care (*P* < 0.0001). Care recipient diagnosis of dementia, poor ability to understand others, and bowel incontinence were also associated with higher CWBI scores for the respective caregivers (*P* < 0.0001). There was a higher proportion of caregivers with higher CWBI scores among those caring for older adults presenting recent (last 90 days) changes in cognition, behaviour and mood (Table 4).

Long-term care home admission of care recipients was predicted by caregiver CWBI scores in logistic regression models when only caregiver characteristics were included (model 1) and also after including only care recipient characteristics in the model (model 2) (Table 5). The goodness of fit of the models including caregiver characteristics only (model 1) or care recipient characteristics only (model 2) improved when both models were merged indicating that caregiver and care recipient characteristics are needed to better predict care recipient LTCH admission (model 3) (Table 5).

**Table 5:** Logistic regression models for selected outcomes associated with care recipient long-term care home admission

Independent variable	Parameter estimate (SE)	Odds ratio (95% CI)	*P* value	C statistict
*Model 1. Caregiver characteristics*				
CWBI^a^ (0–6 vs. 7–8)	1.41 (0.45)	4.13 (1.68–10.17)	0.002	0.73
Age group (65–74 vs. under 65)	0.25 (0.54)	1.28 (0.44–3.71)	0.64	
Age group (75–84 vs. under 65)	−0.06 (0.53)	0.94 (0.32–2.68)	0.90	
Age group (85+ vs. under 65)	−1.10 (1.11)	0.33 (0.037–2.96)	0.32	
Gender (female vs. male)	0.34 (0.44)	1.40 (0.58–3.36)	0.44	
Co-reside with care recipient	0.89 (0.46)	2.46 (0.98–6.14)	0.05	
*Model 2. Care recipient characteristics*				
MAPLe 4 vs. 3	2.22 (0.67)	9.29 (2.45–35.2)	0.001	0.77
MAPLe 5 vs. 3	2.09 (0.68)	8.11 (2.12–31.0)	0.002	
Age group (60 to 74 vs. under 60)	0.86 (0.65)	2.36 (0.65–8.55)	0.18	
Age group (75+ vs. under 60)	1.10 (0.64)	3.02 (0.86–10.59)	0.08	
Gender (female vs. male)	1.06 (0.46)	2.90 (1.16–7.23)	0.02	
*Model 3. Caregiver and Care recipient characteristics*				
*Caregiver characteristics*				
CWBI (0–6 vs. 7–8)	1.25 (0.49)	3.52 (1.32–9.34)	0.01	0.84
Age group (65–74 vs. under 65)	0.37 (0.62)	1.45 (0.42–4.87)	0.54	
Age group (75–84 vs. under 65)	0.36 (0.60)	1.43 (0.44–4.69)	0.55	
Age group (85+ vs. under 65)	−1.38 (1.22)	0.25 (0.023–2.74)	0.25	
Gender (female vs. male)	0.12 (0.51)	1.13 (0.41–3.11)	0.80	
Co-reside with care recipient	1.02 (0.54)	2.76 (0.94–8.09)	0.06	
*Care recipient characteristics*				
MAPLe^b^ 4 vs. 3	2.33 (0.73)	10.29 (2.44–43.25)	0.001	
MAPLe 5 vs. 3	2.08 (0.73)	8.03 (1.90–33.83)	0.004	
Age group (60 to 74 vs. under 60)	0.57 (0.72)	1.78 (0.43–7.32)	0.42	
Age group (75+ vs. under 60)	1.12 (0.69)	3.07 (0.78–12.04)	0.11	
Gender (female vs. male)	0.94 (0.53)	2.55 (0.90–7.25)	0.07	

^a^ *CWBI* Caregiver Wellbeing Index, ^b^*MAPLe* method for assigning priority levels

The standardized Cronbach’s alpha value for the CWBI was 0.89 (*n* = 1020) in the replication sample indicating that this tool presents good internal consistency.

## Discussion

The development of the CWBI represents an advance in caregiver research. This study showed not only that this screener is a valid and reliable measure of caregiver wellbeing but also that this measure is directly related to positive and negative aspects of a caregiver’s lives. On the one hand, high scores were linked with negative aspects such as financial concerns, inability to continue in their role, physical pain, and loneliness while on the other hand, lower scores were associated with positive aspects of life such as caregivers feeling valued and respected by others, capable of managing stresses, satisfied with life, and hopeful about their future. A highlight of the validation study was the association between poor caregiver wellbeing and care recipient long term care home admission, changes in their behaviour, and worsening of their mood, decision making, and communication.

The screener differentiates levels of wellbeing linked to positive and negative aspects of caregiver lives. This is in agreement with other studies [64] but it also has other important implications. Since wellbeing has a protective role in health maintenance and poor wellbeing is associated with impaired health [8], the screener may be used to identify caregivers that would benefit from target interventions or preventive measures, using only four items. According to Dodge et al. [6], wellbeing is related to the psychological social, and physical resources that someone has to address as a result of challenges on these aspects of their lives [6]. Thus, caregivers can achieve wellbeing by receiving necessary resources (e.g., information, social support, respite) to maintain a sense of equilibrium.

The association between CBWI scores and caregiver related outcomes shows how psychological wellbeing is related to a caregiver’s perceived health and multiple doctor or nurse appointments. These findings are also consistent with other studies that report high prevalence of poor health among family caregivers [4] and higher health care utilization [65].

It is possible that a substantial number of caregivers, particularly with the higher scores on the CWBI, were experiencing or could be at risk of depression as they were more likely to report loneliness, hopelessness, and daily physical pain. This is concerning considering that depression affects quality of life and may lead to a caregiver contemplating suicide [66] and tragic outcomes, highlighting the need to monitor the mental health of caregivers.

Interestingly, caregivers with excellent/good subjective wellbeing were more likely to present a positive outlook (i.e., ‘feel good about myself’). It is important to note that caregiver life satisfaction has been linked with positive caregiving experiences even when they report high burden [9]. In addition, the presence of a large number of friends and close relationships has been associated with overall life satisfaction of caregivers [67].

The results of the present research clearly demonstrate multidimensionality of the factors related with caregiver wellbeing (e.g., perceived physical health, loneliness, financial concerns, personal outlook). These findings are consistent with interactions between caregiver wellbeing and other aspects of their lives providing evidence on the validity of the screener [58, 64, 68–70].

The validation of the screener using information collected during a follow-up pilot study at a large home care organization emphasized the relationship between different aspects of a caregiver’s life such as their relationship with family (social resource), presence of distress, anger, and depression (psychological resources) and their wellbeing. Caregiver perceived burden or distress has been associated with wellbeing [71] and may affect the ability to continue in the caregiving role. In this study, caregivers with higher CBWI scores (poor wellbeing) were more likely to feel unable to continue. The inability to continue among caregivers with high CBWI scores was emphasized by the association found between high caregiver CWBI scores and the respective care recipient LTCH admission even after controlling for care recipient age and health care needs (i.e., MAPLe). This finding is consistent with studies showing the linkage between caregiver wellbeing and care recipient LTCH admission [48].

The CWBI has the advantage of being fully compatible with interRAI standards for assessments that are widely used in Canada and elsewhere [72]. Linking caregiver information from the pilot survey with the most recent care recipient interRAI CHA or RAI-HC allows for the assessment of caregiver needs at the same time that care recipient health is examined. In this study, the linkage between the RAI-HC assessment of the care recipient and the CWBI indicated some patient characteristics that could be affecting caregiver wellbeing. For example, CWBI scores were not only associated with the health care needs of the care recipient (i.e., MAPLe) but also with care recipient worsening in decision making, communication, changes in behavioural symptoms, and mood decline at the time of the assessment compared to the status of 90 days ago. These associations may indicate the impact of recent changes in symptoms on caregiver psychological wellbeing [6, 73].

The result of standardized Cronbach alpha indicates that the screener is a reliable tool with items measuring a similar construct. The screener scores provide a measure of caregiver wellbeing that is not only driven by care recipient needs but also by caregiving experiences, available resources, and caregiver mental and physical health.

As an observational study, the present research does not allow for making causal inferences. Another limitation is that the screener was developed using information from caregivers that were already experiencing stress and therefore many were likely experiencing poor wellbeing, although the validations sample included caregivers experiencing different levels of wellbeing. The generalizability of the outcomes of this study is limited to caregivers of older adults receiving home care.

There are a number of advantages to using the CWBI. The fact that the CWBI was developed using items common to other interRAI assessments is an important advantage of this tool. The screener can be applied on its own or used in conjunction with an interRAI assessment of the care recipient to facilitate the design of an integrated care plan to address the needs of both persons in the dyad.

The CWBI identifies caregivers at risk of adverse outcomes with a small number of items that could be implemented in a relatively straightforward manner, especially by organizations that assess a high volume of care recipients and caregivers. The CWBI consists of only four questions, focusing in one concept (psychological wellbeing). The CWBI is different than other screeners used in caregiver research such as the risk appraisal measure (RAM), a screener with 16 questions [74] or the Tailored Caregiver Assessment and Referral (TCARE), a 32 question screen used to develop care plans for informal caregivers [75, 76].

The CWBI has been validated using various data sources. Scores of this short screener were associated with caregiver and care recipient characteristics and outcomes (long term care admission) and therefore could be used to flag the need for further assessment of the caregiver to target interventions that match their unique needs.

The CWBI scores were associated with negative and positive aspects of caregiving. Results from this study demonstrate the complexity involved in caregiving by reporting negative and positive aspects of their experiences, the latter often omitted in caregiving studies. This is a novelty compared with most instruments that focus on burden. The choice of using a psychological wellbeing measure acknowledges that caregiving may result in both positive and negative feelings, where unstable feelings affect the screener scores. Moreover, this measure seems a better predictor of self-reported health than other caregiver measures [77].

## Conclusion

This screener has the potential to identify caregivers that would likely benefit from further assessment and intervention (such as respite care) with the goal of improving their quality of life. For example, clinical treatment may be appropriate for caregivers affected by depression or pain, whereas caregivers experiencing loneliness might benefit from participation in a support group. Further research to explore the outcomes of using the CWBI in other jurisdictions, coupled with information from the interRAI CHA or RAI-HC, has the potential to provide key information to be used by policy and decision makers when developing strategies and funding initiatives to better support caregivers in their role.

## Abbreviations

CIHI: Canadian Institute for Health Information
CRS: Caregiver Recharge Services
CSI: Caregiver Strain Index
CWBI: Caregiver Wellbeing Index
HNHB: Hamilton Niagara Haldimand Brant
LHIN: Local Health Integration Network
LTCH: long term care home
MAPLe: Method of Assigning Priority Levels
MH: Mississauga Halton
SAS: Statistical Analysis System
